# Physical Fitness and Somatic Characteristics of the Only Child

**DOI:** 10.3389/fped.2020.00324

**Published:** 2020-06-25

**Authors:** Luis P. Rodrigues, Ricardo Franco Lima, Ana Filipa Silva, Filipe Manuel Clemente, Miguel Camões, Pantelis Theodoros Nikolaidis, Thomas Rosemann, Beat Knechtle

**Affiliations:** ^1^Escola Superior Desporto e Lazer, Instituto Politécnico de Viana do Castelo, Rua Escola Industrial e Comercial de Nun'Álvares, Viana do Castelo, Portugal; ^2^Research Center in Sports Sciences Health Sciences and Human Development, CIDESD, Vila Real, Portugal; ^3^N2i, Polytechnic Institute of Maia, Maia, Portugal; ^4^Instituto de Telecomunicações, Delegação da Covilhã, Portugal; ^5^Exercise Physiology Laboratory, Nikaia, Greece; ^6^Institute of Primary Care, University of Zurich, Zurich, Switzerland; ^7^Medbase St. Gallen Am Vadianplatz, St. Gallen, Switzerland

**Keywords:** only child, motor competence, motor performance, human development, children

## Abstract

The purpose of this study was to examine if only child show differences on somatic growth and physical fitness compared to be a child with siblings. The participants included 542 children (boys: *N* = 270; girls: *N* = 270) between 7 and 15 years of age. Somatic growth (height, weight, body mass index) and physical fitness (handgrip strength; flexed harm hang; 60-s sit-ups; standing long jump; 10-m shuttle run and PACER test) were assessed. Variance analysis revealed significant advantages for children with siblings in the flexed arm hang (*p* = 0.046), 60-s sit-ups (*p* = 0.002), 10-m shuttle run (*p* = 0.013) and PACER (*p* = 0.032). An examination of the possible differential effect of sex on the results revealed no significance for physical fitness variables, but significant interaction were found for weight (*p* = 0.004) and body mass index (*p* = 0.005). Despite a lack of interactions between offspring and sex in physical fitness, significant differences between sexes were found in all fitness variables. In conclusion, having siblings showed to be advantageous for general physical fitness in children. This evidence may be used for future analysis and interventions in motor competence, namely considering the growing number of only children in some regions of the world.

## Introduction

In the European Union in 2016, almost half (47% or 31 million) of all households with children had only one child. In Portugal, there was also a tendency for single-child families to increase over the last few decades. In 1991, 44% of Portuguese households with children had only one child. This number rose to 51% in 2001, and to 55% in the last 2011 census. At the same time, the percentage of couples with three or more children has decreased (17, 11, and 8% in 1991, 2001, and 2011 respectively), and the number of couples with two children has remained at 38% (1). Surely this phenomenon of the only child, associated with the diminishing autonomy of children and young people (2), has an effect on the opportunities (affordances) for motor stimulation in these children and, consequently, on the development of their motor competence and physical fitness. For instance, solitary play, without a brother or sister, will be more sedentary, focused on individual play without movement, while the lack of autonomy will hamper children from experiencing new environments and motor challenges, with evident implications on his(her) perceive motor competence and self-confidence.

The only-child condition was widely examined in the literature throughout the 20th century and was particularly boosted by the single child policy imposed in China and by the general concern that the development of only children could be impaired by a lack of stimulation from siblings (3).

On the contrary, the expectation of an enriched family-child involvement and investment (4) within the context of only-child education and development conflicts with previously stated ideas. In an extended review from 1986, Fabo and Polit showed that English-speaking only children had more positive developmental outcomes (achievement, character, and intelligence) than their peers with siblings.

Urban Korean only children showed a greater tendency for depression (5), while Brazilian only sons were less likely to have an alcohol intoxication episode during adolescence (6).

In a recent study with 20,592 adult subjects in New Zealand, Stronge et al. (3) tested for differences in Big Six personality traits in adults and found that the ones with no siblings showed lower average levels of honesty-humility and conscientiousness and higher levels of neuroticism and openness. However, while statistically significant, these differences did not rise to the level of practical effects (3).

Physical fitness is a determinant of healthy child development, as it is related to several health outcomes and is a good summative measure of the body's ability to perform physical activity and exercise (7, 8).

Children with low levels of physical fitness (9) and motor competence are at greater risk for obesity (10).

A significantly higher likelihood of being overweight and obese has been found in only children, both in a recent systematic review and meta-analysis (11) and amongst a national sample of 43,046 children born in 2001 in Japan (12). Children with no siblings also had significantly lower levels of moderate-to-vigorous physical activity than children with siblings (13).

It is largely reported in research that environmental variables are important in influencing positive health behaviors and improving physical fitness. Several studies have revealed that birth weight, the mother's lifestyle during her pregnancy, the father's health, and the presence of siblings had the strongest influence on children's fitness (14–18). Regarding the birth order of siblings, the literature shows differences in motor development between older and younger siblings (15). These differences are probably due to the influence of older siblings on younger ones, although the authors conclude that this relationship may depend on biological characteristics.

The presence of siblings and peers seems to be a predictor of enriched motor development (19, 20). When the sibling influence is compared for sportsmen, elite athletes are more likely to be later-born children, while non-elite athletes are more likely to be the firstborn (21).

Although the number of single-child households is increasing, the consequences for child motor development have not been fully addressed in the literature. Individual pathways of change in physical fitness and growth are expected to be influenced by children's immediate environments and the presence or absence of other children in the family.

Consequently, the aim of this study is 2-fold: (i) to examine if being an only child is associated with negative differences on somatic growth and physical fitness compared to being a child with siblings and (ii) to analyze whether these differences are influenced by the child's sex. Our specific hypothesis is that only child will show detrimental differences in physical fitness and weight status.

## Materials and Methods

### Sample

Participants in this study belong to the Melgaço Youth Observatory (MYO), a mixed-longitudinal growth and development project that is currently taking place at this location in the north of Portugal. A convenience sample that included all participants who entered the study between 2015 and 2019 was selected. A database was organized with data from the first year of assessment of each MYO participant, resulting in a total of 542 children (270 boys; 270 girls), aged from seven to 15 years of age (boys mean age= 10.47 ± 2.67; girls mean age=10.44 ± 2.64). Within the sample, 141 children were only child (71 boys; 70 girls) and 399 children had brothers or sisters (197 boys, 202 girls).

### Procedures

The study was approved by the Scientific Council of the Polytechnic Institute of Viana do Castelo with the reference CTC-ESDL-001-2014. School directors approved the study, adult participants and the parents or tutors of underage children gave their informed consent. Children also gave verbal assent prior to data collection. All procedures were carried out in accordance with the 1964 Helsinki declaration and its later amendments.

Participants were individually interviewed by a research assistant to fill a sociodemographic questionnaire containing information about the family (parent's professional occupation, number of brothers and sisters and respective age). All somatic characteristics and physical fitness assessments were done in the same order at the laboratories of the Melgaço School of Sports and Leisure. Observers were trained in the assessment's protocols and its specifications, and each observer was responsible for only one test or measure. At least two of the three first authors of this study personally supervised all data collection.

### Assessments Protocols

#### Somatic Measures

Somatic measurements included height and weight, which were measured with a SECA 217 stadiometer and a SECA 762 weight scale. All measurements were taken according to the International Society for the Advancement of Kinanthropometry's (22) standardized protocol.

Body mass index (BMI) was calculated using by dividing body weight (in kilograms) by height (in square meters).

#### Physical Fitness

Handgrip strength (HS) was tested using the handgrip dynamometer (SAEHAN, model SH5001), with individuals seated with their shoulders adducted, their elbows flexed at 90°, and their forearms in a neutral position, according to the American Society of Hand Therapists (23). In this analysis, we reported data only for the right hand, as some children of younger ages were not fully able to report their preferred hand.

Flexed arm hang (FAH) performances were assessed by noting the time the participant could hold herself with the chin above the bar, arms flexed, and using a supine grasp position. Two observers helped the child to assume the initial position, and the stopwatch counted the time between this moment and the moment the child's chin touched the bar or fell below the level of the bar. Results are recorded in tenths of seconds.

The maximum number of correct sit-ups (SU) performed in 60 s was counted. The participant started by lying down on a mat with their arms crossed across the chest and legs flexed at ~45°. An observer secured the participant's feet using two hands throughout the test. A correct sit-up was counted when the participant touched their knees with their arms kept close to the chest. The total number of correct SU is the result of the test.

Standing long jump (SLJ) performances were assessed by recording the length of a landing horizontal jump, with the participant departing from a line in the ground at a two-foot-long take-off. The result of the test was the best result of three trials. The length of the jump was measured from the departing line to the nearest point where the heels touch the ground. The results were recorded in cm.

Performances in a 10-m shuttle run (SHR) were recorded using the following protocol. Two parallel lines were marked on the floor 10 meters apart. Two blocks of wood were placed behind one of the lines opposite the starting line. On the signal “Ready? Go!” the child ran to the blocks, picked one up, ran back to the starting line, and placed the block behind the line; he then ran back and picked up the second block, which he carried back across the starting line. Two attempts were allowed, with the best time used as the result of the test.

The PACER test is a widely used progressive test where participants run back and forth at a specified pace from two lines that are 20 meters apart. The pace is externally regulated by an auditory sign (a beep) that marks the moment participants should be at each end of the course (a lap). The pace is increased every minute, and participants remain in the test until they can no longer keep up with the pace at the end of two consecutive laps. Participants were encouraged to achieve their maximal performance. When they did not, according to the observer's judgment (e.g., when showing a lack of motivation to complete the task, stopping due to injury or pain, or not showing facial flushing, sweating, hyperpnoea, or an unsteady gait), the result was not included. For children below 10 years of age, a pacing light apparatus was used throughout the testing time to assure full participation and motivation in the 20-m SRT test. The number of completed laps was recorded as the result of the test.

#### Maturation

Time to Peak Height Velocity (PHV) was used as a maturational index assessed according to the following equations for each sex (24):

Time to PHV Boys = −8.3971103 + (0.0070346 ^*^ decimal age ^*^ sitting height).

Time to PHV Girls = = −7.709133 + (0.0042232 ^*^ decimal age ^*^ height).

### Statistics

Descriptive statistics for age groups (7–9, 10–12, 13–15 years-of-age) according to the sex and offspring condition are presented for all variables. A two factor ANCOVA full factorial model was used to test for the effects of Sex (boy or girl) and Offspring (only child or sibling) on each somatic and physical fitness variable, while adjusting for decimal age (covariate). All variables were previously tested for normality and homoscedasticity and, in accordance, the FAH and PACER data were logarithmic transformed. Residuals configurations from of the ANCOVAs were scrutinized for possible non-normal or biased configurations.

## Results

Descriptive results according to sex, age, and existence of siblings in the family can be found in Table 1.

**Table 1:** Number of subjects, mean, and standard deviation values for all variables according to age group, sex, and the existence of siblings in the family.

		**Girls**				**Boys**			
		**Only child**		**Sibling**		**Only child**		**Sibling**	
**Variable**	**Age**	***n***	**Mean SD**	***n***	**Mean SD**	***n***	**Mean SD**	***n***	**Mean SD**
Height	7–9	36	129.8 ± 6.7	110	130.3 ± 6.6	32	133.0 ± 7.3	107	130.3 ± 7.4
	10–12	19	150.6 ± 10.5	46	148.8 ± 9.4	23	147.4 ± 8.7	45	143.9 ± 6.7
	13–15	15	158.4 ± 5.5	46	158.1 ± 6.3	16	165.4 ± 5.9	45	165.6 ± 10.0
Weight	7–9	36	29.8 ± 6.9	110	30.3 ± 6.9	32	34.1 ± 10.8	107	30.5 ± 8.4
	10–12	19	43.9 ± 11.4	46	42.6 ± 9.2	23	45.0 ± 12.0	45	40.4 ± 9.9
	13–15	15	53.2 ± 8.9	46	56.8 ± 10.2	16	61.3 ± 11.3	45	55.9 ± 13.5
BMI	7–9	36	17.6 ± 3.2	110	17.7 ± 3.0	32	18.9 ± 4.3	107	17.7 ± 3.4
	10–12	19	19.1 ± 3.4	46	19.1 ± 2.8	23	20.4 ± 3.9	45	19.3 ± 3.4
	13–15	15	21.2 ± 3.4	46	22.8 ± 4.3	16	22.3 ± 3.5	45	20.2 ± 3.2
HG	7–9	36	12.3 ± 2.5	110	12.3 ± 3.0	32	13.2 ± 3.4	107	13.0 ± 3.1
	10–12	19	21.1 ± 6.0	46	19.9 ± 5.0	23	18.9 ± 6.0	45	20.5 ± 5.1
	13–15	15	24.2 ± 4.5	46	25.2 ± 3.8	16	32.6 ± 6.2	45	31.8 ± 7.4
FAH	7–9	36	7.8 ± 6.3	110	11.0 ± 10.8	32	9.0 ± 11.2	107	10.9 ± 8.3
	10–12	19	8.2 ± 8.6	46	7.3 ± 9.4	23	9.0 ± 9.2	45	16.5 ± 17.4
	13–15	15	14.1 ± 10.9	46	12.8 ± 10.2	16	37.0 ± 21.3	45	43.2 ± 24.4
SU	7–9	36	23.0 ± 9.7	110	25.2 ± 8.1	32	24.3 ± 9.0	107	26.4 ± 7.2
	10–12	19	27.9 ± 6.8	46	30.5 ± 6.8	23	29.4 ± 9.8	45	34.4 ± 8.2
	13–15	15	32.7 ± 4.0	46	33.1 ± 7.0	16	40.9 ± 8.7	45	43.5 ± 8.9
SLJ	7–9	36	108.6 ± 15.6	110	113.3 ± 17.9	32	114.7 ± 20.3	107	117.6 ± 19.0
	10–12	19	133.6 ± 23.5	46	128.6 ± 18.6	23	125.9 ± 22.2	45	139.4 ± 20.1
	13–15	15	135.5 ± 20.2	46	130.4 ± 21.3	16	179.9 ± 27.9	45	176.0 ± 24.4
SHR	7–9	36	14.5 ± 1.2	110	14.2 ± 1.2	32	14.1 ± 1.4	107	13.8 ± 1.4
	10–12	19	12.7 ± 1.0	46	12.7 ± 0.9	23	13.0 ± 1.5	45	12.2 ± 1.2
	13–15	15	12.4 ± 0.7	46	12.5 ± 1.0	16	10.7 ± 0.9	45	10.8 ± 0.8
PACER	7–9	36	22.7 ± 8.4	110	25.3 ± 9.8	32	25.8 ± 12.5	107	30.2 ± 16.0
	10–12	19	28.5 ± 12.6	46	36.6 ± 16.1	23	37.0 ± 18.8	45	41.2 ± 18.6
	13–15	15	29.6 ± 10.0	46	30.3 ± 11.4	16	66.1 ± 20.8	45	61.7 ± 18.5

Although no differences were found between decimal ages of boys and girls (*p* = 0.521), girls proved to be maturational advanced relative to boys (time PHV = −1.37 ± 2.19 and −2.78 ± 1.97 respectively for girls and boys; *p* < 0.001). Nonetheless no differences were found in the time to PHV between the only child and the siblings' groups (*p* = 0.500). The correlation between decimal age and maturational time to PHV was of 0.98 for boys and 0.99 for girls showing that decimal age is also a very good indicative of both chronological and biological age of the participants.

In order to understand if being an only child can result on deleterious differences on the somatic growth and physical fitness of children, a two-way ANCOVA was run for each collected variable. The main effect of interest was related to the offspring condition of being an only child compared with having other siblings in the house, but we were also interested in understanding if being a boy or a girl can affect this possible offspring effect. Since different ages were present in the sample, we used decimal age as a covariate in order to control for the age and maturation effect in the variables. The results are shown in Table 2 below.

**Table 2:** Main effects for Offspring condition, Sex, and interaction between them, controlling for decimal age, for each somatic and physical variable.

	**Offspring**		**Sex**		**Interaction**	
	**Only child (yes or no)**		**(boy or girl)**		**Sex x Offspring**	
	***F***	***sig***	***F***	***sig***	***F***	***sig***
**Somatic growth**						
Height	4.062	***p=*** **0.044**	7.578	***p=*** **0.006**	1.897	*p =* 0.169
Weight	4.316	***p=*** **0.038**	6.006	***p=*** **0.001**	8.251	***p=*** **0.004**
BMI	2.414	*p =* 0.121	1.832	*p =* 0.177	7.896	***p=*** **0.005**
**Physical Fitness**						
Handgrip	0.025	*p =* 0.873	19.636	***P<*** **0.001**	0.178	*p =* 0.673
Flexed arm hang	3.989	***p=*** **0.046**	9.145	***p=*** **0.001**	1.579	*p =* 0.209
60 s Sit-ups	9.355	***p=*** **0.002**	21.714	***P<*** **0.001**	0.379	*p =* 0.539
Standing Long Jump	1.265	*p =* 0.261	40.907	***P<*** **0.001**	1.399	*p =* 0.237
10 m SHR	6.166	***p=*** **0.013**	35.809	***P<*** **0.001**	0.910	*p =* 0.340
PACER	4.636	***p=*** **0.032**	33.958	***P<*** **0.001**	0.075	*p =* 0.784

*Decimal age was a covariate present in all statistical tests reported in the table, and showed a significant effect for all models (p < 0.001). Significant values (p < 0.05) are showed in bold*.

For all tested variables, except for BMI, Sex has proved to have a significant effect on the outcome value, as expected. Similar thing happened with Decimal Age entering as a covariate in the models, but in this case the effect was significant for all variables (*p* < 0.001 for all models).

Interaction between Offspring conditions and Sex was never significant for all physical fitness variables tested but turned out as significant for Weight and BMI, meaning that the Offspring effect on weight and BMI can be different depending on the sex of the child.

**Figure 1:**
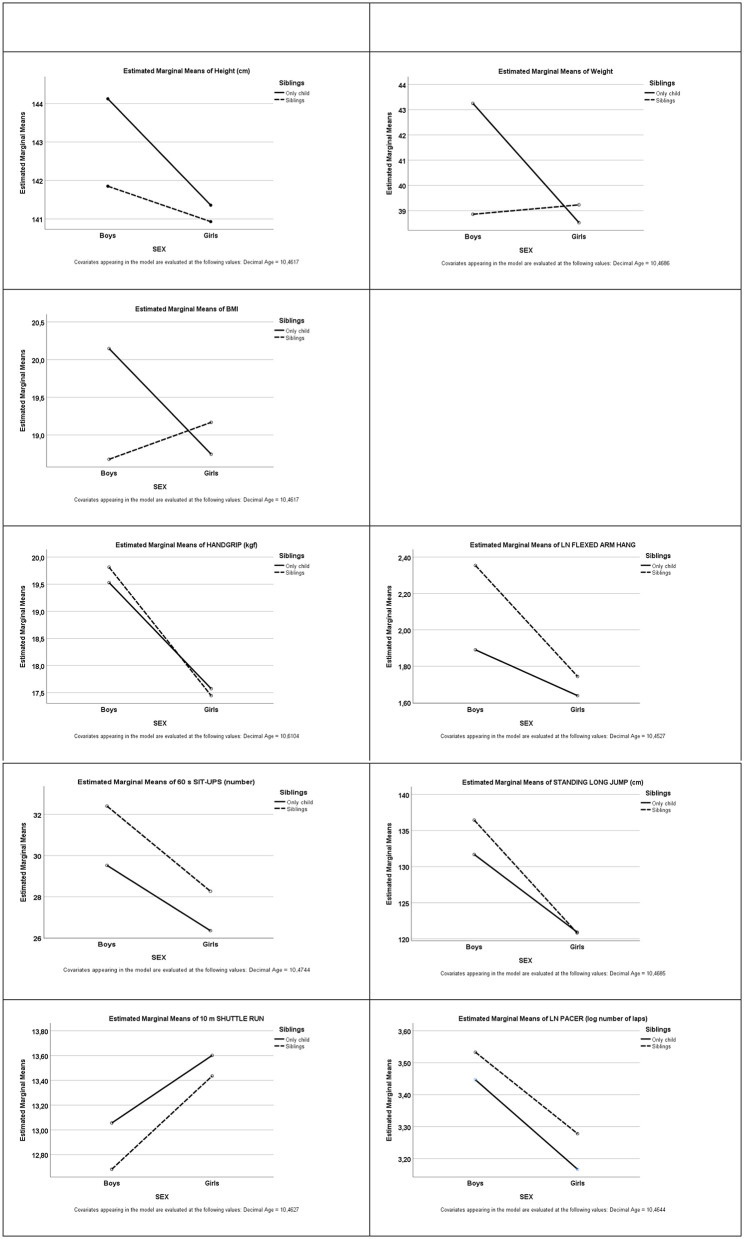
Representation of the estimated marginal mean for somatic measures and physical fitness tests according to the number of siblings (only child or with siblings) by sex.

Finally, and looking for our condition of interest in this study, the Offspring condition, we can see that significant differences were found between children that are the only child in the family and the ones that have brothers and/or sisters (siblings) for most of the physical fitness variables (FAH, SU, SHR, and PACER), and for height and weight. In general, the difference found was deleterious for the only child group that always showed a worst average performance in the physical fitness tests but tend to be taller and heavier than the sibling's group (see Figure 1).

## Discussion

The aim of the present study was to understand whether being an only child can be associated with a deleterious difference on somatic growth and physical fitness between the ages of seven and fifteen, our hypothesis being that those differences will be identified in physical fitness and weight status.

Our results showed that only children were taller (*p* = 0.044) and heavier (*p* = 0.038) than children with siblings, although no differences were found in BMI (*p* = 0.121). The growth and maturity features of a child (morphological, physiological, and neuromuscular) have an important role in the development of motor performance during infancy and childhood (25). Differences in height and weight during late childhood and adolescence can be due to differences in maturational status (25–28). However, in the present case, no differences were found in maturational time at PHV (peak height velocity) between these two groups. Genetics and nutritional or environmental conditions are possible explanations for this phenomenon, but more information is needed to substantiate these allegations.

Considering weight, different profiles were observed between sexes regarding each child's condition, with boys achieving higher results in the only-child group and girls achieving higher scores in the children with siblings group. The boys' results were in line with a study conducted with Chinese children aged six to eighteen, which showed that only children were about four times more likely to be obese than children with siblings, even after controlling for sex, age, parental weight status, parental education level, household income, and urban/rural residence (29). Also, Bagley et al. (30) found that boys without siblings spent more time watching television than boys with siblings. The consequence of this was an increase in sedentary time and, in turn, an increase in body weight. On the other hand, the girls' results could be related to the fact that having a sibling has been associated with a 2-fold increase in the likelihood of adolescents viewing ≥ 2 h of television per day (31). Nevertheless, contradictory results were found in the same study that registered less time spent viewing television in girls with siblings compared to girls without siblings. In the same vein, the BMI results displayed a different profile regarding sex and offspring conditions, although any sex differences were registered.

After removing the effect of age, children with siblings showed better results in four of the six tested items (FAH, 60 s sit-ups, 10 m SHR, and PACER), with no differences found for the other two items (handgrip and SLJ).

Consistent with the literature [e.g., (19, 32–35)], boys performed better than girls in all physical fitness tests across all ages. In fact, studies have shown that girls outperform boys only in tasks that include mainly balancing, hopping (19, 32), and flexibility (36). Such results have been interpreted as a social rather than genetic influence in childhood, as the physical characteristics of girls and boys are very similar (37–39). During pubertal age, sexual dimorphism explains the male's advantages in most physical fitness tasks (37).

Age of the study participants ranged from pre-pubertal to pubertal ages, surely living in different motor skill development periods and maturational levels, both wich can influence physical fitness performance and be associated with sex differences. Trying to account for this question we had sex groups with similar age (10.47 ± 2.67 and 10.44 ± 2.64, respectively, for mean and standard deviation of boys and girls) in the sample. Furthermore, age groups within sexes also showed similar decimal ages (see Table 1) reducing the chance for impacting the main effect of interest in the study. Maturation is expected to influence physical fitness performance but, in this case, decimal age and maturational age (age at peak of height velocity) was found to be highly correlated (0.98 and 0.99, respectively, for boys and girls). Because decimal age was used as a covariate in the analysis, the results are independent for both age and maturation level. Boys and girls had the same decimal age.

Relative age or season of birth, is another known variable that seems to be associated with differences in motor competence specifically in sports, with athletes born in the first months of the year showing advantages on the long term sport's career (40). Our sample showed similar distribution of birth month between only child and non-only child by sex (Boys χ^2^(268,11) = 10.86, *p* = 0.455; Girls χ^2^(272,11) = 10.65, *p* = 0.473), showing that this characteristic did not affect our results.

Muscular fitness is an important marker of health that has been inversely and independently associated with insulin resistance, clustered cardiometabolic risk, and inflammatory proteins during childhood and adolescence (41–43). Considering the strength-related tests conducted in this study, it seems that maximal force (expressed in the HG test) showed no differences between offspring conditions, as well as the leg power test (expressed in the SLJ), in each sex. Nevertheless, in the resistance strength tests (SU and FAH), different profiles were expressed when comparing offspring conditions. In boys, those with siblings clearly demonstrated an advantage, presenting consistently higher values than only-child boys. In fact, other studies suggest that children may benefit from having siblings, especially older siblings who serve as role models, as parents tend to be over-protective of only or firstborn children (19). The same pattern was clearly observed in girls in the SU test, but in FAH, a mixed profile was expressed, with sibling girls showing higher values between 7 and 9 years of age while only child girls outperformed those with siblings in terms of physical growth. Indeed, Wrotniak et al. (44) also concluded that motor proficiency was not related among siblings, while Costa et al. (45) and Pereira et al. (46) observed that only children and firstborn siblings showed greater strength values while exhibiting worse velocity and flexibility results. Such contradictory results confirm the need to conduct more studies in this field.

SHR is commonly used to measure agility (47). In our sample, this capacity differed based on offspring conditions; however, it led to a clear difference between boys and girls. In only-child boys, the 10-m SHR times were slower, especially for those between 7 and 12 years of age. However, in children between 13 and 15 years old, this difference seemed to disappear. It should be noted that the participants in our sample live in a rural environment, which could lead only children to explore the surrounding spaces to a greater extent as they become older and more autonomous. Nevertheless, in girls, this same pattern was observed as being smoother. Only slight differences were observed for girls between 7 and 9 years of age, with the same values maintained in the older ages. Earlier stabilization in girls could be related to the maturation process, which also happens earlier in girls (generally, when they are 10–12 years old) (48).

The PACER, which is strongly associated with health, was developed as a field-based measure for estimating cardiovascular fitness (7, 49). This capacity seems to be developed further in children with siblings than in only children—for both sexes, only children showed poorer results for all age groups, except for boys between 13 and 15 years old. In fact, (50) have suggested that siblings have an unequivocal advantage in motor competence and physical fitness independent of age, sex, or birth order. This phenomenon was observed in children of both sexes of 7 to 15 years of age.

This was the first study that looked at the condition of being an only child in relation to physical fitness. In summary, our results suggest that not having brothers or sisters to play with in the family is a clear disadvantage for the development of physical fitness and highlights the need to conduct more studies in this field, especially because Portuguese statistics show that only-child families are increasing (1).

Although siblings share, on average, 50% of their genes identical-by-descent and a common family environment, they differ in their chronological age, sex, and health behaviors as well as in their physical growth, biological maturation, and motor development trajectories (28). The literature is not consistent when considering who has the advantage between the firstborn [e.g., (17, 51, 52)] or the later-born child [e.g., (15, 19, 53)], suggesting that differences in motor competence and physical fitness exist between siblings depending on birth order and sex.

Very young (preschool age and younger) siblings can spend more time interacting with each other than with any other person, including their parents (54–56). Normally, parents are more protective of an only child, which can result in restrained autonomy and less moving around physically. The only child has the parents' attention for a longer time. In this sense, the family environment seems to play an important role in the development of physical fitness.

In this study, we did not account for the sex, age difference, or the number of siblings in the household. Although including participants from different growing ages, we should keep in mind the cross-sectional nature of the study, so caution should be used when longitudinal inferences are made. Future studies should include these questions and attempt to further disentangle possible causes for the differences found. Age difference and sex of the sibling(s) relative to the child can be variables of interest to analyze in next studies with larger samples. Parenting styles can be of importance, along with the socioeconomic background of the family since there is a known association with the number of siblings. Motor competence as a foundational cornerstone for movement abilities should also be examined within the scope of only children. Longitudinal follow-ups of children, or simply to look for the association of adult's level of motor competence with their offspring condition can probably bring us a better understanding of the only-child motor development phenomena.

Our findings give support to the need for special attention of parents, educators and coaches to the only child physical fitness and somatic development. Parents should try to compensate for the lack of peers in the household by organizing more shared time with friends at home and out of it. Educators should be aware of these potential characteristics of the only child and its consequences within the children's group relationships, and act accordingly. Coaches are to understand the uniqueness of the only child to give them the opportunity to catch up on their specific motor competence whenever the case.

In this study we conclude that children with siblings show positive differences on somatic growth and physical fitness compared to the only child. This conclusion holds for both sexes and for all ages between 7 and 15 years of age.

## Conclusions

The present study compared the somatic characteristics and physical fitness of only children with those of children with siblings. It revealed significantly better levels of flexed arm hang, 60-s sit-ups, 10-m shuttle run, and PACER results in children with siblings despite only children having significantly greater values of height and weight. No significant interaction was found between offspring and sex, considering physical fitness. Comparisons between sexes revealed that boys had significantly better results than girls for the handgrip strength, flexed arm hang, 60-s sit-ups, standing long jump, 10-m shuttle run, and PACER tests. Generally, our results highlight the importance of offspring in the physical fitness of children. This should be carefully considered by parents, educators and specialists who aim to analyze the contexts that may influence motor competence in the future.

## Data Availability Statement

The raw data supporting the conclusions of this article will be made available by the authors, without undue reservation.

## Ethics Statement

The study was approved by the Scientific Council of the Polytechnic Institute of Viana do Castelo. Written informed consent to participate in this study was provided by the participants' legal guardian/next of kin.

## Author Contributions

All authors listed have made a substantial, direct and intellectual contribution to the work, and approved it for publication.

## Conflict of Interest

The authors declare that the research was conducted in the absence of any commercial or financial relationships that could be construed as a potential conflict of interest.
